# Serum magnesium is inversely associated with coronary artery calcification in the Genetics of Atherosclerotic Disease (GEA) study

**DOI:** 10.1186/s12937-016-0143-3

**Published:** 2016-03-01

**Authors:** Rosalinda Posadas-Sánchez, Carlos Posadas-Romero, Guillermo Cardoso-Saldaña, Gilberto Vargas-Alarcón, María Teresa Villarreal-Molina, Nonanzit Pérez-Hernández, José Manuel Rodríguez-Pérez, Aida Medina-Urrutia, Esteban Jorge-Galarza, Juan Gabriel Juárez-Rojas, Margarita Torres-Tamayo

**Affiliations:** 1 Departamento de Endocrinología, Instituto Nacional de Cardiología Ignacio Chávez, Juan Badiano 1, Sección XVI, Mexico City, 14080 Mexico; 2 Departamento de Biología Molecular, Instituto Nacional de Cardiología Ignacio Chávez, Juan Badiano 1, Sección XVI, Mexico City, 14080 Mexico; 3 Instituto de Medicina Genómica, Laboratorio de Genómica de Enfermedades Cardiovasculares, Periférico Sur 4809, Arenal Tepepan, Mexico City, 14610 Mexico

**Keywords:** Serum magnesium, Coronary artery calcification, Subclinical atherosclerosis

## Abstract

**Background:**

Serum magnesium is inversely associated to coronary artery calcification (CAC) in patients with chronic kidney disease. There is little information on this association in a general healthy population.

**Objective:**

The aim of this study was to examine the cross-sectional association of serum magnesium levels with CAC.

**Methods:**

We included 1276 Mexican-mestizo subjects (50 % women), aged 30–75 years, free of symptomatic cardiovascular disease. CAC was quantified by multidetector computed tomography using the method described by Agatston. Cross-sectional associations of serum magnesium with cardiometabolic factors and subclinical atherosclerosis defined as a CAC score > 0, were examined in logistic regression models adjusted for age, sex, education, smoking status, body mass index, systolic blood pressure, physical activity, elevated abdominal visceral tissue, fasting insulin and glucose, alcohol consumption, menopausal status (women only), low (LDL-C) and high density lipoprotein cholesterol (HDL-C), triglycerides, diuretic use, type 2 diabetes mellitus (DM2), and family history of DM2.

**Results:**

After full adjustment, subjects in the highest quartile of serum magnesium had 48 % lower odds of hypertension (*p* = 0.028), 69 % lower odds of DM2 (*p* = 0.003), and 42 % lower odds of CAC score > 0 (*p* = 0.016) compared to those with the lowest serum magnesium. The analyses also showed that a 0.17 mg/dL (1SD) increment in serum magnesium was independently associated with 16 % lower CAC (OR 0.84, 95 % CI 0.724–0.986).

**Conclusions:**

In a sample of Mexican-mestizo subjects, low serum magnesium was independently associated to higher prevalence not only of hypertension and DM2, but also to coronary artery calcification, which is a marker of atherosclerosis and a predictor of cardiovascular morbidity and mortality.

## Introduction

Magnesium plays an important role in many biochemical processes including all ATP transfer reactions. Magnesium is essential for insulin release by pancreatic β-cells, and is also and important second messenger for insulin action [1, 2]. Cross-sectional [3] and longitudinal studies [4, 5] have found a significant inverse association between serum magnesium and DM2, insulin resistance and inflammation. Moreover, magnesium is a natural calcium antagonist [6] and modulates vasomotor tone, blood pressure, and peripheral blood flow [7, 8]. In prospective studies, serum magnesium levels have been inversely associated with the incidence of hypertension [9] and coronary heart disease (CHD) [10]. Increased carotid intima-media thickness (CIMT) and coronary artery calcification (CAC) are both markers of subclinical atherosclerosis, and are also predictors of cardiovascular disease (CVD) morbidity and mortality independently of traditional CVD risk factors [11–13]. Inverse associations between serum magnesium and CIMT have been reported [3, 14]. However, conflicting results have been found in the two studies investigating the association of dietary magnesium and CAC [15, 16]. Moreover, a negative association between serum magnesium and vascular calcification has been observed in patients with chronic kidney disease [17]. The only observational study that has examined this association in a population-based study reported that low serum magnesium level is associated with CAC in Koreans subjects [18]. However, it is known that CAC prevalence and amount are heavily influenced by ethnicity [19, 20]. Therefore, in the present study we assessed the cross-sectional association of serum magnesium concentrations with coronary artery calcification in Mexican-mestizo subjects with no family history of premature CHD and asymptomatic for CVD.

## Methods

### Study population

The study population included participants of the Genetics of Atherosclerotic Disease (GEA) study. The GEA study was designed to examine the genomic bases of CHD and assess relationships between traditional and emerging risk factors with clinical and subclinical atherosclerotic vascular diseases in an adult Mexican-mestizo population [21]. Briefly, a convenience sample of 1200 CHD patients and 1500 control subjects aged 30–75 years was recruited from residents in Mexico City. Patients with established premature CHD were selected from the outpatient clinic of the National Institute of Cardiology. Volunteer control participants with a negative family history of premature CHD and no personal history of cardiovascular disease were recruited from blood bank donors, and through brochures posted in Social Services centers. Coronary patients and control subjects with a history of renal, liver, thyroid, or malignant disease, as well as those on treatment with corticosteroids, were excluded. The GEA study was approved by the Institutional Review Board of the National Institute of Cardiology and conducted according to the Declaration of Helsinki. Written informed consent was obtained from all participants.

This study is a cross-sectional analysis of the baseline measurements of 1500 control subjects included in the GEA study. The participants answered structured questionnaires that provide detailed information regarding family history, demographics, diet, physical activity, medications, smoking, and alcohol intake. We excluded all subjects with missing CAC (*n* = 25), missing physical activity data (*n* = 111), missing dietary data (*n* = 45), missing insulin (*n* = 1) and magnesium sampling (*n* = 42). The final study population included in the analysis comprised 1276 subjects. Systolic and diastolic blood pressures were measured after rest for at least 10 min, and the average of the second and third measurements was recorded for analyses. Hypertension was defined as systolic blood pressure ≥ 140 mmHg or diastolic blood pressure ≥ 90 mmHg or the current use of antihypertensive medication. Height, weight, and waist circumference were measured and BMI was calculated as weight in kilograms divided by height in meters squared. The metabolic syndrome (MS) was defined using the criteria from the American Heart Association/National Heart, Lung, and Blood Institute scientific statement on the MS [22], except for central obesity that was considered when waist circumference was > 90 cm in men and > 80 cm in women [23]. Type 2 diabetes mellitus (DM2) was defined by the American Diabetes Association criteria [24], reported glucose-lowering treatment or previous DM2 diagnosis by a physician. Family history of DM2 was defined as either mother or father having DM2. Insulin resistance was estimated using the homeostasis model assessment (HOMA-IR). Insulin resistance was defined as HOMA-IR values ≥ 75th percentile (3.66 in women and 3.38 in men). These cutoff points were obtained from a GEA study sample of 131 men and 185 women without obesity and with normal of blood pressure, fasting glucose and lipids measurements.

### Biochemical analyses

Venous blood samples were collected from subjects after a 12 h fast. Plasma glucose, total cholesterol, triglycerides, HDL-C, apolipoprotein A (apo A), apolipoprotein B (apo B), creatinine, uric acid, aspartate aminotransferase (AST), and alanine aminotransferase (ALT), were measured in fresh samples, using standardized enzymatic procedures in a Hitachi 902 analyzer (Hitachi Ltd,Tokio, Japan). Accuracy and precision of lipid measurements in our laboratory are under periodic surveillance by the Centers for Disease Control and Prevention service (Atlanta, GA, USA). LDL-C was estimated using the DeLong et al. formula [25]. Total high-sensitivity C-reactive protein (hs-CRP) levels were determined by immunonephelometry on a BN Pro Spec nephelometer (Dade Behring Marburg GmbH, Germany). Interassay coefficient of variation (CV) values were <6 % for all of these assays. Serum total adiponectin was determined by ELISA (R&D Systems, Minneapolis, USA) Quantine kit. The intra- and interassay CV were < 10 %. Plasma insulin concentrations were determined by a radioimmunoassay (Millipore; RIA Kit, Cat. No. HI-14 K, MO, USA) the intra- and interassay CV values were 2.1 and 6.8 %, respectively. Serum magnesium levels were measured by using the direct xylidyl blue complex method in a COBAS C311 automated chemistry analyzer (Roche diagnotics GmbH, Germany). The intra and interassay coefficient of variation were ≤1.0 %.

### Computed tomography study

Visceral adipose tissue (VAT), subcutaneous fat, liver and spleen attenuation, and coronary artery calcium were quantified by computed tomography in each participant. Computed tomography of the chest and abdomen were performed using a 64-channel multidetector helical computed tomography system (Somatom Cardiac Sensation, 64, Forcheim, Germany) and interpreted by experienced radiologists. Scans were read to assess and quantify the following: i) total abdominal, subcutaneous, and VAT areas as described by Kvist et al. [26]; ii) liver to spleen attenuation ratio (L:SAR) as described by Longo et al. [27]; and iii) CAC score using the Agatston method [28]. Subclinical atherosclerosis was defined as the presence of a CAC score > 0.0 and hepatic steatosis as L:SAR ≤1.0 [29].

### Data analysis

Data are expressed as mean ± S.D., median [interquartile range] for continuous variables, or as proportions for categorical variables. Serum magnesium concentrations were categorized by quartile distribution with the lowest quartile serving as the reference. Differences across quartiles were assessed with ANOVA or Kruskal-Wallis test for continuous variables, and Chi square test for categorical variables. Multivariate logistic regression analysis was used to evaluate the association between each S.D. increase in serum magnesium concentrations with coronary risk factors and CAC. For coronary risk factors, the forced-entry logistic regression analyses were adjusted for age, sex, education, smoking status, elevated abdominal visceral adipose tissue, body mass index, systolic blood pressure, low and high density lipoprotein cholesterol, triglycerides, fasting insulin and glucose, physical activity, alcohol consumption, menopausal status (women only) and diuretic use. For CAC, three forced-entry logistic regression models were constructed: an unadjusted model; a model adjusted for age, sex, education, smoking status, body mass index, systolic blood pressure, and physical activity (model 1); a regression model further adjusted for elevated abdominal visceral adipose tissue, fasting insulin and glucose, and alcohol consumption (model 2); and, finally, a regression model adjusted for the variables included in model 2 plus menopausal status (women only), low and high density lipoprotein cholesterol, triglycerides, diuretic use, DM2 and family history of DM2 (model 3). *P* values <0.05 were considered statistically significant. All statistical procedures were performed using the SPSS software (SPSS version 15.0, Inc.).

## Results

This study included 1276 subjects (640 women, 636 men) with a mean age 54 ± 9 (SD) years, with no family history of premature CHD or CVD symptoms. Compared to women, men had a higher prevalence of high blood pressure (11.9 % vs 7.3 %, *p* = 0.006), insulin resistance (61.8 % vs 54.7 %, *p* = 0.011) and metabolic syndrome (49.2 % vs 40.9 %, *p* = 0.003). The prevalence of current smoking (25.1 % of men and 18.9 % of women) and type 2 diabetes mellitus (15 % of men and 13.4 % of women) were not significantly different between genders. Two thirds of women (66.4 %) were postmenopausal. The mean serum magnesium concentration in the whole population was 2.06 ± 0.17 (SD) mg/dl, and was slightly higher in men than in women (2.07 ± 0.16 (SD) mg/dl vs 2.06 ± 0.18 (SD) mg/dl, *p* = 0.09). The overall prevalence of CAC score > 0 was 27.2 %. (41.5 % of men and 13 % of women, *p* < 0.001).

Table 1 shows clinical, computed tomography and laboratory characteristics of participants according to serum magnesium quartiles. Systolic blood pressure, fasting glucose, HOMA-IR and C reactive protein were inversely associated with serum magnesium levels. High serum magnesium levels were also associated with lower prevalence of hypertension, DM2, insulin resistance, metabolic syndrome, and CAC score >0. A similar non-significant trend was observed for body mass index and fasting insulin levels. In contrast, total and LDL-cholesterol and apolipoprotein B were greater with increasing serum magnesium levels. Visceral adipose tissue, triglycerides, and adiponectin were not associated with serum magnesium levels.

**Table 1:** Characteristics of 1276 GEA study participants according to serum magnesium quartiles

	Q1 <1.97 mg/dl	Q2 ≥1.97 to <2.07 mg/dl	Q3 ≥2.07 to <2.18 mg/dl	Q4 ≥2.18 mg/dl	P*
N	326	304	338	308	
Serum magnesium (mg/dl)	1.90 [1.83–1.94]	2.03 [2.00–2.05]	2.11 [2.09–2.14]	2.24 [2.20–2.29]	<0.001
Dietary magnesium (mg)	351 [290–410]	344 [292–421]	356 [289–424]	358 [293–422]	0.692
Male (%)	50	49.3	51.2	49	0.949
Age (years)	54 ± 10	53 ± 10	54 ± 9	55 ± 9	0.079
Body mass index (kg/cm^2^)	28.8 ± 4.2	28.4 ± 4.2	28.0 ± 4.0	28.1 ± 3.8	0.056
Visceral adipose tissue (cm^2^)	157 [112–211]	148 [112–190]	150 [109–194]	148 [115–193]	0.196
Systolic blood pressure (mmHg)	118 [108–130]	113 [103–125]	114 [106–123]	113 [103–123]	<0.001
Glucose (mg/dl)	95 [87–125]	91 [85–99]	89 [83–96]	89 [84–96]	<0.001
Insulin (μU/ml)	18.1 [13.2–24.9]	18.2 [12.6–24.7]	16.7 [12.4–22.8]	16.4 [12.3–22.4]	0.067
HOMA-IR	4.9 [3.0–7.5]	4.2 [2.8–5.7]	3.7 [2.6–5.4]	3.6 [2.6–5.1]	<0.001
Cholesterol					
Total (mg/dl)	190 ± 36	190 ± 37	193 ± 37	198 ± 37^a,b^	0.015
LDL (mg/dl)	116 ± 32	117 ± 33	117 ± 32	123 ± 33^a,b,c^	0.009
HDL (mg/dl)	45.7 ± 13.2	45.1 ± 13.3	47.8 ± 14.1	45.6 ± 13.0	0.065
Triglycerides (mg/dl)	148 [114–200]	152 [113–202]	145 [106–209]	153 [116–204]	0.844
Apolipoprotein B (mg/dl)	92 [74–112]	93 [75–111]	95 [79–115]	103 [85–122]	<0.001
Apolipoprotein A (mg/dl)	133 [114–154]	131 [111–152]	134 [117–158]	135 [117–155]	0.095
Adiponectin (μg/ml)	8.0 [4.8–11.8]	7.6 [5.0–12.1]	8.1 [5.2–13.1]	7.8 [5.0–12.2]	0.915
hsCRP (mg/l)	1.8 [0.9–3.6]	1.5 [0.8–3.3]	1.4 [0.7–2.9]	1.4 [0.8–3.0]	0.018
Current smoking (%)	21	23	23	21	0.765
Physical activity	7.75 [6.88–8.88]	7.88 [6.75–8.75]	7.88 [7.13–8.75]	7.75 [7.00–8.63]	0.256
Menopausal women (%)	62	62.3	64.8	76.6	0.018
Hypertension (%)	14	8	8	8	0.009
Type 2 diabetes mellitus (%)	34	10	8	5	<0.001
Insulin resistance (%)	66	63	53	51	<0.001
Metabolic syndrome (%)	54	44	41	41	0.003
CAC score >0 (%)	35	24	26	24	0.002

Values are expressed as mean ± SD, median [IR, interquartile range] or percentages. *HOMA-IR* Homeostasis model assessment insulin resistance, *LDL* Low density lipoprotein, *HDL* High density lipoprotein, *hsCRP* High sensitive C reactive protein

*for ANOVA, Kruskal-Wallis or Chi square test. *P* <0.05 ^a^versus Q1, ^b^versus Q2, ^c^versus Q3

Multivariate logistic regression analyses were performed to examine the associations of serum magnesium levels with cardiometabolic risk factors and CAC score >0 (Table 2). After adjustment for several confounding factors, individuals with the highest serum magnesium levels (fourth quartile) had 48 % lower odds lower odds of hypertension (*p* = 0.028), 69 % lower odds of DM2 (*p* = 0.003), and 42 % lower odds of CAC score > 0 (*p* = 0.016) as compared to those in the lowest quartile.

**Table 2:** Association of serum magnesium levels with cardiometabolic risk factors and subclinical atherosclerosis

	Q1	Q2	Q3	Q4
		Odds ratio [CI 95 %]	Odds ratio [CI 95 %]	Odds ratio [CI 95 %]
Hypertension	1	0.52 [0.295–0.917]	0.56 [0.323–0.961]	0.52 [0.294–0.931]
Insulin resistance	1	1.26 [0.891–1.940]	0.96 [0.632–1.467]	1.02 [0.694–1.506]
Metabolic syndrome	1	1.04 [0.715–1.512]	1.01 [0.696–1.467]	1.02 [0.964–1.506]
Type 2 diabetes mellitus	1	0.42 [0.199–0.886]	0.34 [0.161–0.705]	0.31 [0.143–0.672]
CAC score >0	1	0.68 [0.436–1.070]	0.78 [0.507–1.197]	0.58 [0.374–0.915]

Data are expressed as odds ratios and 95 % confidence interval (CI) as assessed by multivariate logistic regression analyses. Other covariates included in the multivariable regression model, along with serum magnesium were: age, sex, education, smoking status, elevated abdominal visceral adipose tissue, body mass index, systolic blood pressure, low and high density lipoprotein cholesterol, triglycerides, fasting insulin and glucose, physical activity, alcohol consumption, menopausal status (women only), family history of type 2 diabetes mellitus, type 2 diabetes mellitus and diuretic use

Associations of a 1SD (0.17 mg/dL) increment in serum magnesium levels with hypertension, insulin resistance, metabolic syndrome, and DM2 are described in Table 3. Variables included in the multivariable model were age, sex, education, smoking status, elevated abdominal visceral adipose tissue, body mass index, systolic blood pressure, LDL-C, HDL-C, triglycerides, fasting insulin and glucose, physical activity, alcohol consumption, menopausal status (women only) and diuretic use. Higher serum magnesium was associated with 19 % lower prevalence of hypertension and 38 % lower prevalence of DM2 per each 1 SD increment in magnesium concentration.

**Table 3:** Associations of serum magnesium concentrations with coronary risk factors

	Odds ratio	P
Hypertension	0.81 [0.673–0.985]	0.034
Insulin resistance	0.89 [0.763–1.044]	0.155
Metabolic syndrome	1.03 [0.891–1.189]	0.694
Type 2 diabetes mellitus	0.62 [0.489–0.786]	<0.001

Odds ratios are expressed in terms of per SD 0.17 mg/dl increase in serum magnesium levels

Multivariate logistic regression analyses adjusted for age, sex, education, smoking status, elevated abdominal visceral adipose tissue, body mass index, systolic blood pressure, low and high density lipoprotein cholesterol, triglycerides, fasting insulin and glucose, physical activity, alcohol consumption, menopausal status (women only) and diuretic use

In addition, regression models were used to test the association between CAC and serum magnesium levels as a continuous variable. A 0.17 mg/dl (1SD) increment in serum magnesium concentration was associated with 16 % lower coronary artery calcification (odds ratio 0.84; 95 % confidence intervals (CI) 0.741–0.953, *p* = 0.007) in the unadjusted model. This association proved to be independent of age, sex, education, smoking status, body mass index, systolic blood pressure, and physical activity (model 1; odds ratio 0.84; CI 0.724–0.964, *p* = 0.014). A regression model further adjusted for elevated abdominal visceral adipose tissue, fasting insulin and glucose, and alcohol consumption (model 2; odds ratio 0.86; CI 0.737–0.995, *p* = 0.043) continued to show a significant independent association between serum magnesium concentrations and CAC. Finally, after a full adjustment (model 2 plus menopausal status -women only-, low and high density lipoprotein cholesterol, triglycerides, diuretic use, DM2 and family history of DM2), a 0.17 mg/dL (1SD) increment in serum magnesium concentration remained significantly associated with 16 % lower coronary artery calcification (odds ratio 0.84; 95 % confidence interval (CI) 0.724–0.986, *p* = 0.033).

## Discussion

We report here an inverse association between serum magnesium levels and the prevalence of CAC score >0 in a Mexican-mestizo population with no family history of premature CHD or clinical signs or symptoms of CVD. This association remained significant after adjustment for a range of confounding factors, including age, sex, education, smoking status, visceral adipose tissue, body mass index, systolic blood pressure, low and high density lipoprotein cholesterol, triglycerides, fasting insulin and glucose, physical activity, alcohol consumption, menopausal status (women only), diuretic use, DM2, and family history of DM2. Serum magnesium levels were also independently associated with a lower risk hypertension and DM2. The latter associations are consistent with previous reports showing that both conditions are frequently associated with a magnesium deficient state as assessed by decreased circulating concentrations of serum total or ionized magnesium [4, 9, 30, 31].

In regard to the association of magnesium with subclinical atherosclerosis, only two population-based epidemiologic studies have examined the relation of magnesium intake with coronary artery calcification. The first study was conducted in 5281 MESA (the Multi-Ethnic Study of Atherosclerosis) participants aged 45–84 years and free of clinically apparent CVD, reporting no association between magnesium intake and CAC [16]. The more recent study included 2695 offspring or third generation Framing Heart Study participants free of CVD and reported that higher magnesium intake was associated with lower levels of CAC [15].

Although an association between serum magnesium levels and CAC has been reported in patients with chronic kidney disease [20], to date, only one cross-sectional analysis has examined serum magnesium levels in relation to CAC, in large number of Korean individuals without cardiovascular disease participating in a health examination program [18]. After adjusting for potential confounders, a significant inverse association between CAC and serum magnesium was observed, which is consistent with the findings of the present study. In agreement with these investigations, two decades ago the Atherosclerosis Risk in Communities Study reported an inverse association between serum magnesium and carotid intima-media thickness (CIMT) [3]. More recently, in a population-based study, Hashimoto et. al. reported that low serum magnesium levels were not only significantly and independently associated with greater CIMT, but also with the presence of atherosclerotic plaques [14]. Increased CIMT is also a marker of atherosclerotic disease, and like CAC, is and independent predictor of cardiovascular morbidity and mortality [11, 12]. Collectively, the results of this and other studies [3, 14, 15, 18] suggest independent associations between reduced magnesium dietary intake or low serum magnesium levels with subclinical atherosclerosis defined either as increased CIMT or as coronary artery calcification.

To date, the underlying mechanisms responsible for the associations between serum magnesium and CAC are incompletely understood. The adverse cardiovascular risk profile found in subjects with low levels of serum magnesium (higher prevalence of DM2, hypertension, insulin resistance, and metabolic syndrome) could explain the association. However, in our analysis and in the report of Lee et.al. [18], the associations between low magnesium and coronary artery calcification remained significant after adjustment for these potential confounders. This suggests that additional mechanisms, beyond classic cardiovascular and metabolic risk factors, could be partly responsible. Results from other experimental models support the role of these other mechanisms. Experiments in cultured endothelial cells have demonstrated that magnesium deficiency promotes a proatherogenic phenotype [32]. Moreover, dietary magnesium restriction in animal models resulted in reduced plasma and erythrocyte magnesium levels, which was accompanied by endothelial dysfunction and systemic inflammation, well known factors involved in the atherogenic process. Interestingly, these abnormalities were reverted by magnesium supplementation [33]. These findings are in line with the beneficial effects of magnesium on inflammation and endothelial dysfunction [34], and suggest that this mineral may prevent the onset and progression of atherosclerosis through mechanisms beyond known traditional pathways.

The present study has some limitations. First, the cross-sectional design does not allow to establish causal or temporal relationships between serum magnesium and coronary artery calcification. Second, despite the comprehensive nature of the data which allowed adjustment for multiple risk factors, the possibility of confounding from unknown or unmeasured factors cannot be completely ruled out. Third, our study population solely comprised Mexican-mestizo subjects, and thus our findings may not apply to populations of other ethnic backgrounds.

## Conclusion

The results of this study strongly suggest that lower serum magnesium levels are associated with coronary artery calcification in Mexican subjects free of clinically apparent cardiovascular disease. Confirmation of these results in other populations is required. Additional prospective studies are also needed to determine if hypomagnesaemia predicts the development and progression of coronary atherosclerosis.

## Abbreviations

ALT: Alanine aminotransferase
apo A: Apolipoprotein A
apo B: Apolipoprotein B
AST: Aspartate amonitransferase
CAC: Coronary artery calcification
CHD: Coronary heart disease
CIMT: Carotid intima-media thickness
CV: Coefficient of variation
CVD: Cardiovascular disease
DM2: Type 2 diabetes mellitus
GEA: Genetics of atherosclerotic disease
HDL-C: High density lipoprotein cholesterol
HOMA-IR: Homeostasis model assessment for insulin resistance
hs-CRP: High sensitive C-reactive protein
L:SAR: Liver to spleen attenuation ratio
LDL-C: Low density lipoprotein cholesterol
MS: Metabolic syndrome
VAT: Visceral adipose tissue
